# Bronchoalveolar lavage cell profiles and proteins concentrations can be used to phenotype extrinsic allergic alveolitis patients

**DOI:** 10.1186/s40248-019-0175-6

**Published:** 2019-03-12

**Authors:** Martina Sterclova, Magdalena Smetakova, Ludek Stehlik, Jelena Skibova, Martina Vasakova

**Affiliations:** 1 Department of Respiratory Medicine, Thomayer Hospital, Videnska 800, 140 00 Prague 4, Czech Republic; 2 Department of Pathology and Molecular Medicine, Thomayer Hospital, Videnska 800, 140 00 Prague 4, Czech Republic; 3 Department of Statistics, Institute of Clinical and Experimental Medicine, Videnska 1958/9, 140 00 Prague 4, Czech Republic

**Keywords:** Bronchoalveolar lavage, Extrinsic allergic alveolitis, Interleukin 4 receptor, Matrix metalloproteinase 7, Proteinase associated receptor 2, Prognosis

## Abstract

**Background:**

Extrinsic allergic alveolitis (EAA) patients form heterogenous group with different clinical manifestation and different prognosis. We aimed to determine how to phenotype distinct EAA subgroups. Predictive role of the bronchoalveolar lavage fluid (BALF) IL-4Rα concentration at the time of diagnosis with regard to the clinical behavior in EAA patients was studied.

**Methods:**

Concentrations of MMP-7, IL-4Rα, TNF-α, and PAR-2 were measured in the BALF od 71 EAA patients at the time of diagnosis. Lung functions and outcome data were assessed at 12 months after the diagnosis. Correlations between the BALF protein concentration, cell profile, lung functions and patient outcome were determined.

**Results:**

We found positive correlations between BALF IL-4Rα concentration and BALF eosinophils (*p* = 0,006), negative correlation between IL-4Rα BALF concentration and diffusing capacity (DLco) (*p* = 0,003), negative correlation between IL-4Rα BALF concentration and forced vital capacity (FVC) (*p* = 0,004) and negative correlation between IL-4Rα concentration and BALF lymphocytes (*p* = 0,04). The BALF concentration of IL-4Rα was significantly higher in acute exacerbation patients (*p* = 0,0032) and in patients progressing despite corticosteroid treatment (*p* = 0,04). We observed a positive correlation between MMP-7 BALF concentration and the BALF lymphocytes (*p* = 0.05), negative correlation between the PAR-2 BALF concentration and DLco (*p* = 0.04) and a negative correlation between the BALF TNF-α concentration and DLco (*p* = 0.03).

**Conclusions:**

Specific subgroup of EAA patients with more severe functional impact, distinct BALF cell profile and higher IL-4Rα BALF concentration can be differentiated. Correlations between the BALF concentrations of PAR-2, MMP-7 and TNF-α with clinical parameters may reflect the role of inflammation in the pathogenesis of EAA.

## Background

Extrinsic allergic alveolitis (EAA) continue to attract the interest of medical doctors and scientists because of the lack of efficient treatment in patients with dominant fibrotic impairment. Standard treatment in patients with EAA is based on systemic corticosteroids and immunosuppressive drugs. While some EAA patients respond promptly to corticosteroid treatment, a distinct proportion experience treatment failure and further progression of their disease. This phenomenon may be explained by different clinical, radiological and histopathological phenotypes based on distinct pathogenetic processes involved in lung damage and healing.

Eventhough pathogenesis of fibrotic EAA needs to be elucidated, several cytokines have been shown to play role. Close interactions among four substances, that play role in disease pathogenesis, were observed. Tumor necrosis factor alpha (TNF-α) enhances release of interleukin-4 (IL-4) from mast cells [1]. Exposure to IL-4 triggers matrix metalloproteinase 7 (MMP-7) induced Fas ligand cleavage from bronchial epithelial cells and thus may contribute to airway damage and inflammation [2]. TNF-α together with hypoxia stimulates production of MMP-7 by macrophages and also increases expression of proteinase activated receptor-2 (PAR-2) [3]. Activation of PAR-2 was shown to increase invasivity of various cell types, mainly through MMP production and was shown to downregulate cytokine induced epithelial apoptosis [4].

The present study aimed to determine whether population of EAA patients may be regarded as homogenous or whether phenotypically different patients are included within this group. We measured the concentrations of proteins previously described to play roles in disease pathogenesis and reported to be identifiable in bronchoalveolar lavage fluid (BALF) in patients with EAA and then correlated concentrations of proteins with demographic data (gender, smoking history), lung functions (forced vital capacity (FVC), forced expired volume in 1 s (FEV1), diffusing capacity for carbon monoxide (DLco) and BALF cytology profiles (percentages of alveolar macrophages, lymphocytes, neutrophils and eosinophils).

Furthermore, we were interested in further analyzing alpha subunit of interleukin 4 receptor (IL-4Rα) concentrations in the BALF of EAA patients with regard to the clinical behavior of the disease.

## Methods

### Study design

After signing an informed consent form (approved 6/2015 Ethical Committee of Thomayer Hospital and Institute of Clinical and Experimental Medicine, Prague, Czech Republic), 71 patients were prospectively enrolled in the study (start of study 7/2015 – end of study 3/2017). All of them underwent history assessment, physical examination, blood tests including screening for autoimmune diseases, lung function tests, chest high resolution computed tomography (HRCT) and bronchoscopy with bronchoalveolar lavage and transbronchial biopsy. Cases were discussed at a multidisciplinary team meeting, and the patients in whom a diagnosis could not be established with known data underwent either surgical lung biopsy or cryobiopsy. These cases were discussed at another multidisciplinary meeting to confirm the final diagnosis. The diagnosis of EAA was based on a history of exposure to organic inhalation antigens or laboratory proof of exposure (serum specific immunoglobulins G), BALF lymphocytosis, HRCT and histology findings compatible with EAA, if needed [5]. Mean age of studied population was 61.4 ± 11.0 years, 32 men and 39 women were included. Twenty-nine patients had smoking history, 20 were current smokers and 22 have never smoked.

#### Bronchoalveolar lavage

Bronchoalveolar lavage (BAL) was performed during fiberoptic bronchoscopy under local anesthesia. The bronchoscope was wedged into a segmental bronchus of the middle lobe. Four fractions of 50 ml of lukewarm saline were instilled and, after each instillation, gently aspired. Only samples with a recovery > 50% of the instilled volume of fluid were used (mean recovery per fraction was 55 ± 22%). Portions of the retrieved fluid were measured and then mixed together. Ten milliliters of retrieved fluid were centrifuged (1900 rpm/10 min/room temperature), and smears for cytology evaluation were prepared and stained with hematoxylin-eosin. Slides were evaluated in light microscope at a magnification of 100x, cells were counted in 100 optic fields and percentages of alveolar macrophages, lymphocytes, neutrophils and eosinophils were estimated. Two milliliters of BALF were centrifuged separately (1900 rpm/10 min/room temperature), and the supernatant was gently aspired and stored at − 80 °C for further analysis. Concentrations of MMP-7, IL-4Rα, PAR-2 and TNF-α were measured by ELISA (Clouds and Clone Corp., Houston, USA). ELISA tests were performed according to the manufacturer’s instructions. Detection range for MMP-7 was 0,156–10 ng/ml, IL-4Rα 15,62–1000 pg/ml, PAR-2 0,156–10 ng/ml, TNF-α 15,6–1000 pg/ml. Alveolar macrophages represented 53.4 ± 25.1%, lymphocytes 31.3 ± 26.6%, neutrophils 8.2 ± 9.7% and eosinophils 6.4 ± 9.4% of cells in BALF differential cell count. (Data are expressed as the mean ± SD).

#### Lung functions

The diffusing capacity for CO-transfer factor (DLco) was investigated using a ZAN 300 CO-diffusion instrument (Inspire, Oberthulba, Germany). The DLco was measured using the single-breath method. Values are expressed as a percentage of the predicted values: FVC 79.2 ± 21.0; FEV1 79.8 ± 20.0, DLco 51.2 ± 19.3. (Data are expressed as the mean ± SD).

#### Definition of EAA subgroups according to clinical behavior

Lung functions (FVC, DLco), treatment, and the presence of acute exacerbation and/or death were recorded 12 months after the initial diagnostic evaluation (*n* = 48, FVC 81,2 ± 29,4% e.v., DLco 26,3 ± 23,5% e.v.). Progression of EAA was defined as a significant decline of lung function (10% drop of FVC and/or 15% drop of DLco) or acute exacerbation (*n* = 10) or death (*n* = 2) in a 12-month period [6]. Progression criteria were fulfilled in 14 patients. The enrolled patients received either no pharmacological treatment (avoidance of further exposure recommended) or systemic corticosteroids. Treatment decisions were based on the clinician’s evaluation of the patient at the time of diagnosis. Acute exacerbation was defined according to a previously published definition of IPF exacerbation [7]. Seventeen patients were treated with systemic corticosteroids, and 8 of them progressed despite treatment (Table 1). The clinical behavior of the patients with EAA (progressors and non-progressors) and their response to the treatment were compared (related) to IL-4Rα BALF concentrations.

**Table 1:** Clinical features of progressor and nonprogressor subgroup of EAA patients (according to data gained 12 months after diagnosis)

	Progressors	Nonprogressors
Number of cases	14	34
Clinical features		
Age (years)	64.6 ± 10.5	60.3 ± 10.9
Gender (M/F)	8/6	25/9
Smoking status (N/ES)	4/10	11/33
FVC (% e.v.)	79.1 ± 21.6	76.7 ± 22.8
DLco (% e.v.)	50.2 ± 21.8	51.8 ± 7.0
BALF Lym (%)	15.6 ± 14.7	36.6 ± 27.6
Treatment		
Systemic corticosteroids	8	9
Supportive care only	6	0
Avoidance of inhalation antigen only	0	25

*M* Male, *F* female, *N* Nonsmoker, *ES* Ever smoked, *FVC* Forced vital capacity, *DLco* Diffusion capacity for carbon monoxide, *e.v*. expected value

Definition of progression: significant decline of lung function (10% drop of FVC and/or 15% drop of DLco) or acute exacerbation or death in a 12-month period

### Analysis

The relationships between the BALF concentrations of MMP-7, IL-4Rα, PAR-2 and TNF-α, lung functions and BALF cell percentages and the demographic data of patients were evaluated with Spearman’s rank order coefficient. The relationships between the clinical behavior of the disease and the IL-4Rα BALF concentration at the time of diagnosis were evaluated with the Mann-Whitney U test (acute exacerbation data) and Spearman correlations (progressors vs. non-progressors). *P* values > 0,05 were considered significant.

## Results

### Concentrations of MMP-7, IL-4Rα, PAR-2, TNF- α in BALF in EAA whole group of EAA patients, progressors and nonprogressors

The concentrations of MMP-7, IL-4Rα, PAR-2 and TNF-α in the BALF of patients diagnosed with EAA are summarized in Table 2.

**Table 2:** Bronchoalveolar lavage fluid concentrations of MMP-7, IL-4Rα, PAR-2 and TNF-α in the whole group at the time of diagnosis and in progressor and nonprogressor subgroups

	EAA -whole group *n* = 71	Progressors *n* = 14	Nonprogressors *n* = 34
MMP-7 (ng/ml)	2.26 (0.24–5.37) 2.54 ± 1.77	1.93 (0.59–7.68) 2.72 ± 2.47	2.23 (0.24–5.37) 2.48 ± 1.52
IL-4Rα (pg/ml)	745.3 (0–2365.0) 723.11 ± 716.67	758.0 (32.0–2342.0) 1003.4 ± 863.1	451.0 (0–2365.0) 633.6 ± 648.8
PAR-2 (ng/ml)	827.0 (279.0–10,000.0) 2192.89 ± 2654.1	1163.0 (451.0–7353.2) 1796.7 ± 1947.5	1377.8 (279.0–\.0) 2311.7 ± 2838.1
TNF-α (pg/ml)	2.89 (0–7.58) 2.88 ± 2.82	2.17 (0–5.32) 2.54 ± 2.42	3.45 (0–7.56) 2.91 ± 2.43

*EAA* Extrinsic allergic alveolitis, *MMP-7* Matrix metalloproteinase 7, *IL-4Rα* Interleukin 4 receptor α, *PAR-2* Protease activated receptor 2, *TNF-α* Tumor necrosis factor α. Data are expressed as median (min-max) and mean ± SD

### Correlations of MMP-7, IL-4Rα, PAR-2, and TNF-α in the BALF and demographic parameters, lung function and BALF cell percentages at the time of diagnosis in EAA patients (whole group)

**Fig. 1:**
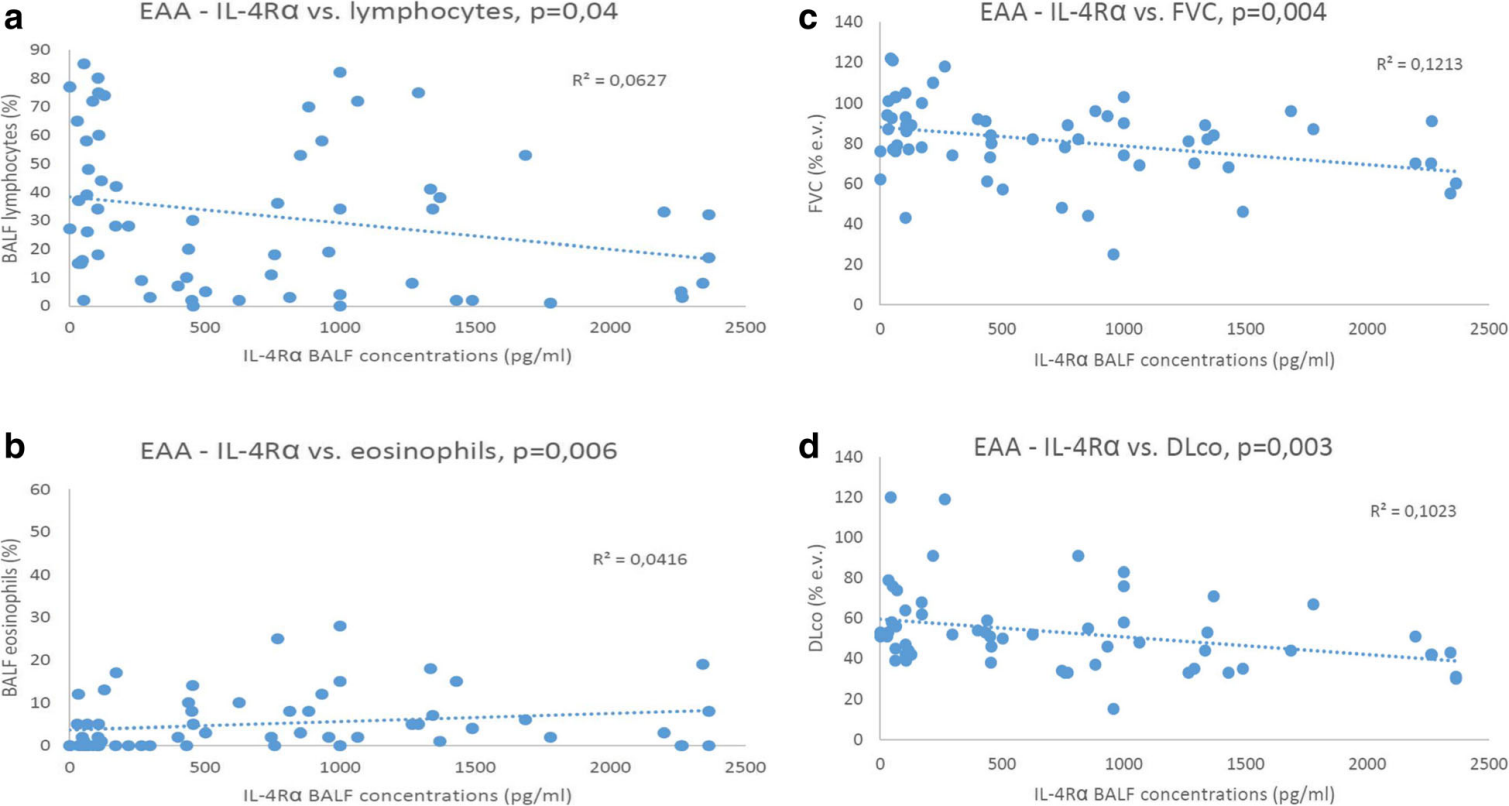
**a**, **b**, **c**, **d** Extrinsic allergic alveolitis – correlations between BALF protein concentrations, DLco, FVC and BALF cell percentages

Significant correlations among BALF proteins, demographic data, lung functions and BALF cell percentages in EAA patients are shown in Fig. 1.

Moreover, we observed a positive correlation between the MMP-7 BALF concentration and the BALF lymphocyte percentage (*p* = 0.05), a negative correlation between the PAR-2 BALF concentration and the DLco (% expected value) (*p* = 0.04) and a negative correlation between the BALF TNF-α concentration and the DLco (% expected value) (*p* = 0.03) in EAA patients.

### Correlations between the clinical behavior of disease in EAA patients and the BALF concentration of IL-4Rα

The BALF concentration of IL-4Rα was significantly higher in patients who experienced acute exacerbation during the observation period (*n* = 10, IL-4Rα = 1480,8 ± 768,0 pg/ml) than in the rest of the group (*n* = 38, IL-4Rα = 656,6 ± 671,6 pg/ml) (*p* = 0,0032). Moreover, the IL-4Rα concentration was significantly higher in patients who progressed despite corticosteroid treatment (*n* = 8; IL-4Rα = 820,6 ± 795,0 pg/ml) compared to the treated and stable group (*n* = 9; IL-4Rα = 714,7 ± 914,0 pg/ml) (*p* = 0,04). Differences in the BALF IL-4Rα concentration between progressors and non-progressors despite any treatment were not statistically significant.

## Discussion

The current study aimed to determine whether we may regard population of patients with EAA to be homogenous or whether this group includes phenotypically different subgroups of patients according to mediators of fibroproliferative healing in BALF. We further studied the predictive role of BALF IL-4Rα concentrations at the time of diagnosis with regard to the clinical behavior of the disease in the subgroup of patients with EAA.

IL-4Rα forms receptors for IL-4 (type I receptor: IL-4Rα + common γ chain, type II receptor: IL-4Rα + IL-13Rα1) and IL-13 (IL-4Rα + IL-13Rα1; IL-13Rα2 – nonsignaling decoy receptor). IL-4Rα mediates the inflammatory response and eosinophil recruitment [8, 9] and our results strongly support this statement showing negative correlation between BALF IL-4Rα concentrations and BALF lymphocyte counts and positive correlation between IL-4Rα concentrations and BALF eosinophil counts (Fig. 1).

Both IL-4 and IL-13 are considered as markers of Th2 cytokine milieu, which is supposed to play role in fibrogenesis in several diseases including EAA [10]. Th2 biased environment favours epithelial mesenchymal transition in chronic EAA, which may result with secretion of further profibrotic substances [11]. We suppose that patients with low FVC and DLco and low BALF lymphocyte counts have chronic EAA with more pronounced fibrogenesis (lymphocytosis was reported mostly in patients with acute/subacute forms of the disease) [5], and the mechanisms leading to fibrogenesis in these patients might be different then in IPF patients, even though the outcome is very similar. Previously shown significant difference of IL-4Rα BALF concentrations in EAA patients compared to other fibrotic interstitial lung diseases (namely IPF) strongly supports this hypothesis [12].

Suspected role of IL-4Rα in EAA pathogenesis together with its easy detectability in BALF made us to further evaluate its possible role as biomarker of treatment response in EAA patients. The present study shows that baseline BALF IL-4Rα concentrations are higher in EAA patients who progressed despite pharmacological treatment (*p* = 0.04). We found also higher baseline IL-4Rα concentrations in patients who experienced acute exacerbation during the 12-month observation period (*p* = 0,0032). According to our previous results, in chronic EAA patients IL-4Rα BALF concentrations were positively correlated with the extent of lung fibrosis on HRCT [13]. Lung fibrosis, namely, the presence of traction bronchiectasis, was found to be a marker of poor prognosis [14, 15]. We suppose that acute exacerbation in these subjects occurs despite treatment, which suggests that treatment with systemic corticosteroids is not able to prevent acute exacerbation of the disease or that acute exacerbation occurs because of corticosteroid treatment. Livraghi et al. found that depletion of IL-4Rα in a mouse model leads to changes in mucus production and reduces mucus plug formation [13]. The administration of prednisolone in this IL-4Rα-depleted mouse model led to a decrease of lymphoid infiltrates and eosinophilia but failed to ameliorate mucus plugging. This finding slightly favored the hypothesis that corticosteroid treatment cannot prevent acute exacerbation; however, more data are needed. Moreover, the protective role of low IL-4Rα concentrations should be explored.

As mentioned above, there are several crosslinks among IL-4, PAR-2, TNF-α and MMP-7 biology and all of them are connected to fibrosis and inflammation. PAR-2 was implicated to play a role in the coagulation cascade, but it was also detected in EAA patients [12]. PAR-2 expression can be upregulated on lung neutrophils after inhalation injury, was confirmed in alveolar epithelial cells, and positively correlates with the extent of honeycombing on HRCT [16, 17]. José RJ et al. concluded that the contribution of PAR-2 to fibrogenesis is highly dependent on both the nature of the insult and disease status [18]. The negative correlation of the PAR-2 concentration in the BALF of EAA patients and the DLco value may reflect the fact that exposure to organic dusts, which are believed to lead to EAA development in susceptible subjects, leads to a PAR-2-dependent inflammatory response [19]. Either fibrotic or inflammatory changes may lead to DLco impairment in this subgroup of patients.

MMP-7 is a profibrotic factor that contributes to the pathogenesis of various lung diseases [20]. It is expressed by fibrocytes, endothelial cells, fibroblasts, myofibroblasts, epithelial cells and leukocytes [21]. It functions to degrade the extracellular matrix and other pericellular substrates, including the adherens junction protein E-cadherin, to promote wound healing and tissue remodeling [22]. The positive correlation between the BALF MMP-7 concentration and the lymphocyte percentage in EAA patients (*p* = 0,05) may reflect tight connection between inflammation and fibrosis in EAA patients.

Although TNF-α is mainly a proinflammatory cytokine, it has been found in lung tissues of patients with fibrotic lung diseases as well. TNF-α is released by alveolar macrophages in EAA patients [23], and the negative correlation between the TNF-α BALF concentration and the DLco value in EAA populations may reflect the role of inflammation in the pathogenesis of the disease.

## Conclusions

The present data suggest that populations of EAA patients are not phenotypically homogenous. Distinct subgroups may be defined by the IL-4Rα BALF concentration both at the time of diagnosis and with regard to the clinical behavior of the disease. Correlations between the BALF concentrations of PAR-2, MMP-7 and TNF-α with clinical parameters in EAA patients may reflect the role of inflammation in the pathogenesis of this disease.
